# Electroactive γ-Phase, Enhanced Thermal and Mechanical Properties and High Ionic Conductivity Response of Poly (Vinylidene Fluoride)/Cellulose Nanocrystal Hybrid Nanocomposites

**DOI:** 10.3390/ma13030743

**Published:** 2020-02-06

**Authors:** Erlantz Lizundia, Ander Reizabal, Carlos M. Costa, Alberto Maceiras, Senentxu Lanceros-Méndez

**Affiliations:** 1 Department of Graphic Design and Engineering Projects, Bilbao Faculty of Engineering, University of the Basque Country (UPV/EHU), 48013 Bilbao, Spain; 2 BC Materials, Basque Center Centre for Materials, Applications and Nanostructures, UPV/EHU Science Park, 48940 Leioa, Spain; ander.reizabal@bcmaterials.net (A.R.); alberto.maceiras@outlook.es (A.M.); senentxu.lanceros@bcmaterials.net (S.L.-M.); 3 Centro de Física, Universidade do Minho, 4710-057 Braga, Portugal; 4 Centro de Química, Universidade do Minho, 4710-057 Braga, Portugal; 5 IKERBASQUE, Basque Foundation for Science, 48013 Bilbao, Spain

**Keywords:** cellulose nanocrystals, nanocomposites, PVDF, electrical properties, mechanical properties

## Abstract

Cellulose nanocrystals (CNCs) were incorporated into poly (vinylidene fluoride) (PVDF) to tailor the mechanical and dielectric properties of this electroactive polymer. PVDF/CNC nanocomposites with concentrations up to 15 wt.% were prepared by solvent-casting followed by quick vacuum drying in order to ensure the formation of the electroactive γ-phase. The changes induced by the presence of CNCs on the morphology of PVDF and its crystalline structure, thermal properties, mechanical performance and dielectric behavior are explored. The results suggest a relevant role of the CNC surface −OH groups, which interact with PVDF fluorine atoms. The real dielectric constant ε’ of nanocomposites at 200 Hz was found to increase by 3.6 times up to 47 for the 15 wt.% CNC nanocomposite due to an enhanced ionic conductivity provided by CNCs. The approach reported here in order to boost the formation of the γ-phase of PVDF upon the incorporation of CNCs serves to further develop cellulose-based multifunctional materials.

## 1. Introduction

Poly (vinylidene fluoride) (PVDF) is a thermoplastic fluoropolymer with large potential in the fields of sensing, actuation, energy storage, harvesting systems, and drug delivery, mainly thanks to its electroactive properties (ferro, pyro and piezoelectric) [1,2,3,4]. This semicrystalline polymer can be found in five different polymorphs (α, β, γ, δ and ε), with the α-phase being the most common polymorph. Traditionally, β-phase PVDF has represented the most technologically relevant phase as it provides a net dipole moment and the largest piezoelectric response among polymers, making PVDF useful for many multifunctional applications [5]. Thus, the development of β-phase PVDF has been intensively pursued in recent years [2]. As melt-processing usually yields the α-phase, PVDF films comprising the β-phase are commonly obtained upon the stretching of the α-phase [6]. Other techniques have been reported so far to effectively promote the formation of such a β-phase, including annealing [7], poling (a preferred orientation of the dipoles along the field direction is induced) [8], and filler addition [9,10].

Interestingly, the γ phase of PVDF is another polar and electrically active phase of PVDF that has been less used for applications [11]. This phase has been typically obtained through the solid-state phase transformation (α–γ) at temperatures that are close to the melt temperature or upon the addition of different fillers such as clays [12]. Filler addition represents a versatile approach to trigger the different crystalline phases of PVDF, as it usually also encompasses the improvement of mechanical, thermal and electrical properties of the matrix. Moreover, no extensive use of equipment is required since fillers can be directly incorporated during the fabrication process, resulting in a cost and time effective approach [2].

In this framework, the addition of fillers such as carbon nanotubes (CNTs) [13], barium titanate (BaTiO_3_) [14], or montmorillonite within the PVDF matrix has been proved to be an efficient approach to enhance the piezoresponse behavior of PVDF [15]. Therefore, a suitable approach to develop PVDF-based materials with improved functionalities may be the introduction of novel organic materials that act as nucleating agents could lead to specific crystalline phases. Cellulose nanocrystals (CNCs) are attractive candidates as they have been proven to prompt the crystallization process of many semicrystalline polymers [16,17,18,19]. CNCs are crystalline, rod-shaped nanoparticles that can be obtained upon the controlled cleavage of amorphous regions of cellulose [20]. These nanoparticles, which can be disintegrated under composting conditions [21], present a high Young’s modulus of 130 GPa [22] and can be synthesized from any cellulose source material by using chemical or enzymatic approaches [20]. The large abundance of cellulose, its mechanical stability at relatively high temperatures [23], low cost, and non-toxicity make CNCs interesting reinforcing elements to improve the piezoelectric properties of PVDF while lowering the environmental burden associated with the use of traditional plastics [24,25].

Apart from being renewable, wood is known to present a piezoelectric effect [26], although its highly heterogeneous, non-crystalline structure usually results in a relatively small piezoelectric constant. In contrast to wood, CNCs present a highly crystalline structure. Since asymmetric crystalline structures have been proven to show a piezoelectric response, the development of such highly crystalline particles makes CNCs, by themselves, an electroactive material with interesting electroactive properties [27]. The high-aspect ratio of CNCs may help to induce the different conformations to PVDF, as their provided large surface-area could serve as heterogeneous nucleation sites of PVDF [28], while the large amount of available –OH groups on the surface of CNCs may interact with PVDF fluorine atoms to yield a net dipolar moment.

Though several works have reported on the preparation of PVDF/cellulose blends [29,30,31], none of them have focused on the effective nucleation of the electroactive phases of PVDF while using CNCs. Therefore, here CNCs are introduced with different contents up to 15 wt.% into PVDF in order to induce morphological changes and nucleate the electroactive phases of PVDF due to available –OH groups on the surface of CNCs. The influence of CNC incorporation into PVDF on the morphology, thermal stability and mechanical performance of PVDF is also investigated, and these nanocomposites can be used in new applications considering natural reinforcements based on wood.

## 2. Materials and Methods

### 2.1. Starting Materials

Microcrystalline cellulose (310697-500G), sulphuric acid, and dimethylformamide (DMF) were obtained from Sigma Aldrich, while poly (vinylidene fluoride) (PVDF) with a molecular weight of *M_w_* = 300 kg·mol^-1^ was purchased from Solvay (Brussels, Belgium) under the name of Solef^®^ 6010.

### 2.2. CNCs Synthesis and their Transfer from Water to DMF

Cellulose nanocrystals (CNCs) are commonly obtained after a chemically-induced cleavage of cellulose by using acid hydrolysis. Different acids including hydrochloric, nitric, hydrobromic and phosphoric acids can be used, although sulphuric acid-induced extraction is the most commonly found method [32,33,34]. Accordingly, CNCs were synthesized here by hydrolyzing 20 g of microcrystalline cellulose into 400 mL of 64 wt.% sulphuric acid solution at 45 °C for 30 min under 300 rpm of magnetic stirring [19]. The reaction was quenched by adding 10-fold distilled water, and the remaining suspension was centrifuged at 4000 rpm for 15 min to remove the excess of acid. Nanosized cellulose was obtained after sonication (Vibracell Sonicator, Sonics and Materials Inc., Danbury, CT) at 50% output for 15 min. For further purification, the suspension was dialyzed over 7 days by using Visking dialysis membranes (molecular weight cut off: 12.000–14.000 Da; Medicell Membranes Ltd. London, UK). Water-dispersed CNCs were exchanged to DMF via centrifugation by using acetone as an intermediate solvent. DMF-dispersed CNCs were stored at 4 °C.

### 2.3. Nanocomposite Fabrication

PVDF/CNC nanocomposites with CNC concentrations of 1, 5, 10 and 15 wt.% were obtained through solvent-casting followed by quick vacuum drying. After dissolving 500 mg of 10 mL PVDF in DMF, the required amount of CNC (up to a maximum of 88 mg for the 15 wt.% nanocomposite) was added to obtain nanocomposites with different concentrations. After dispersion with a magnetic stirring plate (Ika, model no. C-MAG HS 7) and sonication (Vibracell Sonicator, Sonics and Materials Inc., Danbury, CT, USA), the mixtures were cast onto Petri-dishes (ø 70 mm), and the DMF was removed in 15 min with a vapor chamber under vacuum from J.P. Selecta S.A.U. (Barcelona, Spain) at 70 °C [2,35]. For comparison, we also fabricated films upon the evaporation of DMF at 60 °C at atmospheric pressure for two weeks (see Figure S1 for its morphological, conformational, thermal and mechanical properties). Films that were 50 ± 5 μm thick were obtained (and measured with a digital micrometer).

### 2.4. Characterization

CNC morphology was evaluated by transmission electron microscopy (TEM) in a Philips CM120 Biofilter apparatus (Koninklijke Philips N.V. Amsterdam, Netherlands). A droplet of a 0.1 wt.% water-dispersed CNCs was deposited onto a carbon-coated grid and was then negatively stained with 1% uranyl acetate for 1 min.

PVDF/CNC nanocomposite morphology was examined by using a scanning electron microscope (SEM, NanoSEM—FEI Nova 200 (FEG/SEM)) at an accelerating voltage of 15 kV. Before analysis, cryogenically-fractured surfaces were coated with a gold layer by sputtering with a Polaron SC502 apparatus.

The attenuated total reflectance Fourier transform infrared spectroscopy (ATR-FTIR) of CNCs was performed on a Bruker Alpha FT-IR Spectrometer equipped with diamond ATR optics (Jasco FT/IR-4100 system). FTIR spectra were obtained from 4000 to 600 cm^−1^ with 64 scans having a resolution of 4 cm^−1^.

The X-ray powder diffraction (XRD) patterns were recorded on a PANalytical Empyrean diffractometer in reflection mode by using Cu Kα radiation (λ = 1.5418 Å) and operating at 45 kV and 40 mA.

The CNC zeta-potential at room temperature was measured in a Malvern Zetasizer Nano-ZS using diluted aqueous CNC dispersions (0.1 mg·mL^−1^).

Thermal transitions were analyzed by using differential scanning calorimetry (DSC), while thermal stability was investigated through thermogravimetric analysis (TGA). DSC was carried into a Netzsch DSC 204 F1 Phoenix instrument under a flowing nitrogen atmosphere between 25 and 200 °C at a rate of 10 °C ·min^−1^ (for both cooling and heating). All samples were measured in 40 µL aluminum pans with perforated lids. TGA was performed in a TGA Q500 (TA Instruments) between 30 at 700 °C at 20 °C·min^−1^ under a N_2_ flow of 50 mL·min^−1^.

The mechanical behavior of the PVDF nanocomposites was evaluated under tensile testing by using an Autograph AGS-J from Shimadzu with 20 mm × 50 mm plain jaws, vise grips, a 20 mm gauge, and a 100 N load cell. Samples were conditioned at 22 °C and 51% relative humidity overnight before testing. Stress–strain curves were obtained at a rate of 5 mm·min^−1^ for samples being 5 mm in width, 25 mm in length, and 50 μm in thickness. The Young’s modulus (*E*) value was obtained from the slope in stress–strain curve within the initial linear elastic region, i.e., from the slope between 0.5% and 1.5% strain in the stress–strain curves. Tests were performed according to the ASTM D882-18 standard (Standard Test Method for Tensile Properties of Thin Plastic Sheeting) for sample thicknesses below 1 mm (reported values were determined as the mean value of 5 samples) [36].

Dielectric measurements at room temperature were carried out with a Quadtech 1920 Precision LCR Meter in the 20 Hz–1 MHz frequency range with an applied voltage of 0.5 V. Five millimeter diameter aluminum electrodes were vacuum evaporated onto both sides of each sample. The error associated with the dielectric measurements is about 2%. The real part of the dielectric function (*ε’*), the dielectric losses (*tan δ*) and real part of the conductivity function (*σ’*) were obtained according to:

(1) $$\varepsilon^{\prime }=\frac{C.d}{\varepsilon_{0}.A},$$

(2) $$\tan\delta =\frac{\varepsilon^{\prime \prime }}{\varepsilon^{\prime }},$$

(3) $$\sigma^{\prime }=\varepsilon_{0}\omega \varepsilon^{\prime \prime },$$

where *A* indicates the plate area, *d* is the plate distance, *ε_0_* (8.85 × 10^−12^ F·m^−1^) is the permittivity of free space, *ε’* and *ε’’* are the real and imaginary dielectric constants, respectively, and ω = 2πν is the angular frequency.

## 3. Results and Discussion

### 3.1. CNC Characterization

**Figure 1:**
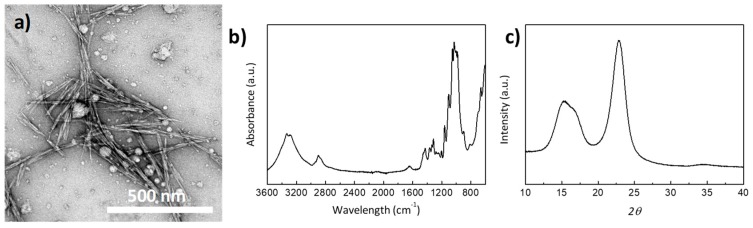
(**a**) Representative TEM images, (**b**) FTIR spectrum, and (**c**) XRD pattern of cellulose nanocrystals (CNCs).

Here, we developed PVDF/CNC nanocomposites through solvent casting and vacuum fast drying in order to induce the nucleation of the γ-phase of PVDF towards enhanced electroactive properties. Synthesized CNCs presented a rod-shaped morphology with a length of 135 ± 21 nm and a width of 7 ± 1 nm (see Figure 1a). The FTIR results showed the characteristic features for cellulose, with a broad band in the 3650–3200 cm^−1^ region arising from the characteristic O–H stretching vibration and narrower bands in the 1500–800 cm^−1^ region; the most indicative ones were those centered at 13337, 1160 and 897 cm^−1^ due to the C–O–H bending, C–O–C bending and C–O–C asymmetric stretching, respectively. Additionally, three main diffraction peaks at 2*θ* = 14.7°, 16.4° and 22.7°, respectively, in the XRD pattern were observed, suggesting the presence of cellulose I [37,38]. The crystallinity index (CI) of CNCs could be extracted from the XRD pattern in Figure 1c following [39]:

(4) $$CI=\frac{A_{c}}{A_{c}+A_{a}}\;\times \;100,$$

where *A_c_* and *A_a_* account for the total crystalline and amorphous areas, respectively. The amorphous area can be extracted after an XRD pattern deconvolution based on Ruland’s principles and Rietveld analysis). Accordingly, CNCs with a CI of 93% were obtained.

As a result of the sulphuric acid-assisted hydrolysis, CNC surfaces are decorated with sulphate half-ester groups, yielding negatively charged surfaces with an average zeta potential (ζ-potential) of −39.6 ± 0.9 mV. Further evidence of the sulphate half-ester groups was observed in the FTIR absorption band located at 1033 cm^−1^ (see Figure S2). This negatively charged character of CNC surfaces may serve to induce an electroactive phase nucleation in PVDF as positively charged CH_2_ groups within the PVDF interact with sulphate half-ester groups onto CNCs to yield an extended all-trans TTTT conformation [1,10]. This conformation corresponds to the all-trans conformation of PVDF and is formed due to the fact that CNC surfaces promote the crystallization of the PVDF chains into all-trans conformation through the interaction of sulphate half-ester groups with the dipole moments of PVDF [10].

### 3.2. Morphology and Structure of PVDF/CNC Nanocomposites

**Figure 2:**
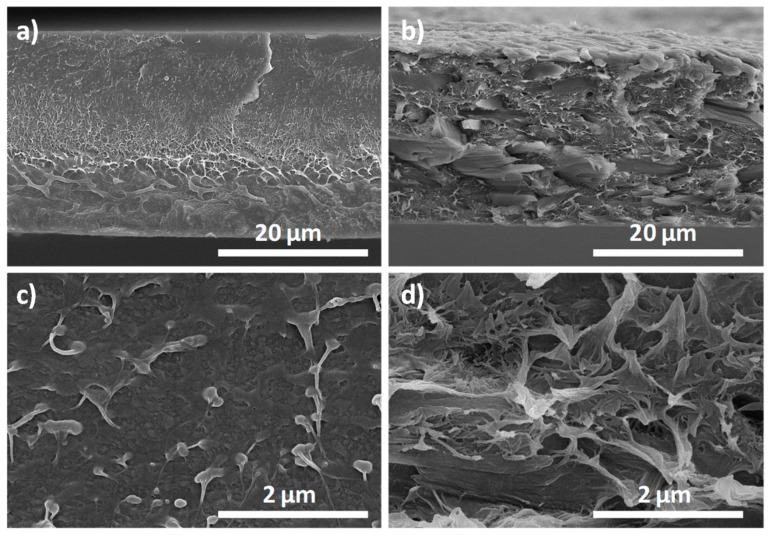
SEM micrographs of cryogenically fractured poly (vinylidene fluoride) (PVDF) (**a** and **c** at higher magnification) and PVDF/CNC 10 wt.% nanocomposite surfaces (**b** and **d** at higher magnification).

PVDF/CNC nanocomposite morphology was firstly assessed with scanning electron microscopy (SEM) to provide insights on the morphology of PVDF and the degree of CNC dispersion [9]. Figure 2 shows SEM micrographs of cryogenically fractured PVDF (Figure 2a,c) and PVDF/CNC (Figure 2b,d) surfaces. As observed in both low and high magnification SEM images (Figure 2a,c), neat PVDF presents a relatively smooth fracture surface with some scattered spherulites [9,28]. This morphology markedly differs from the one that was obtained upon drying at 60 °C under atmospheric pressure (Figure S1a), which failed to provide the electroactive γ-phase. Upon CNC addition, the fracture surface became notably rougher, suggesting that the presence of CNCs prompted PVDF crystallization. In spite of the obvious scale difference, no traces of CNC bundles could be observed in the high-magnification SEM image in Figure 2d, suggesting that, a priori and irrespectively of the CNC–PVDF interfacial strength, no large CNC aggregates were formed during sample preparation [40].

**Figure 3:**
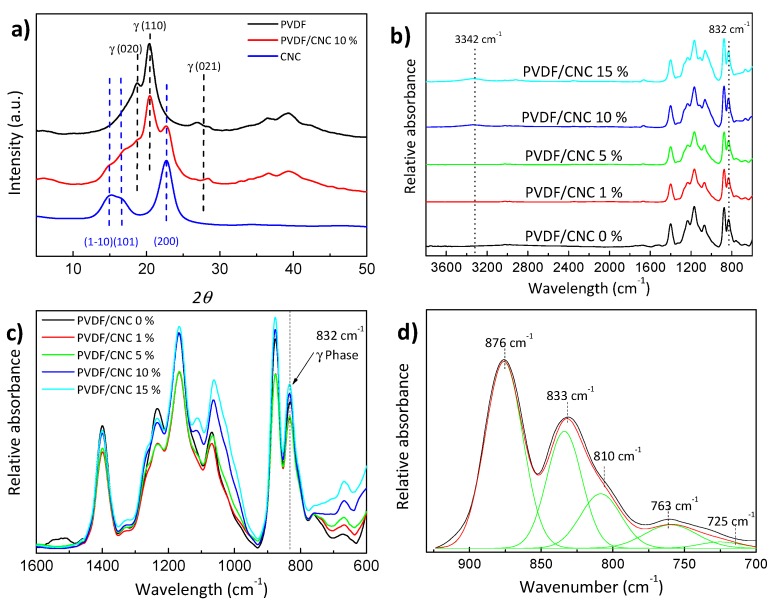
(**a**) Wide angle X-ray diffraction (WAXD) patterns; (**b**) FTIR spectra and (**c**) enlarged FTIR spectra of the PVDF/CNC nanocomposites and (**d**) peak deconvolution of the FTIR spectra in the 925–700 cm^−1^ region for PVDF.

The occurrence of different phases in PVDF nanocomposites can be studied by X-ray diffraction and Fourier transform infrared spectroscopy (FTIR). Accordingly, Figure 3 presents wide angle X-ray diffraction (WAXD) patterns and FTIR spectra for the PVDF/CNC nanocomposites with CNC concentrations up to 10 wt.%. The three peaks located at 2*θ* = 14.9°, 16.5° and 22.7° corresponded to the (1-10), (101) and (200) planes of the cellulose I phase [38,41]. The PVDF and PVDF/CNC nanocomposites displayed three peaks located at 18.7°, 20.4° and ~27°, which corresponded to the *(020), (110)* and *(021)* planes that are characteristic of the γ-phase [12].

The presence of the γ-phase of PVDF was also confirmed through the FTIR spectra shown in Figure 3b–d (see Figure S1b for the FTIR spectrum of the film dried at 60 °C under atmospheric pressure). The specific bands for each nanocomposite constituent in the PVDF/CNC nanocomposites are summarized in Table 1.

For neat PVDF, the specific characteristic bands are 766, 796, 855 and 976 cm^−1^ for α-PVDF, 840 and 1275 cm^−1^ for β-PVDF, and 812, 833, 838 and 1234 cm^−1^ for γ-PVDF. Though it is difficult distinguish the peaks related to the β-phase and the γ-phase in the 800–900 cm^−1^ region, the specific bands corresponding to the γ-phase could be observed at 431, 812 and 1234 cm^−1^ [42]. The specific band characteristics of the α-phase at 766 cm^−1^ and the γ-phase at 812 and 833 cm^−1^ are identified in the Figure 3b,c for all PVDF/CNC nanocomposites, while Figure 3d shows the peak deconvolution for the PVDF (the specific bands for the α and γ-phases are highlighted). As shown in Figure 3c, the obtained spectra did not change with the CNC concentration. Therefore, we estimated that the main reason for γ-phase nucleation was the vacuum application during sample preparation, where the solvent quickly evaporated to yield the γ-phase. Typically, in a conventional oven at temperatures below 90 °C, the evaporation rate of DMF is low, i.e., a lower polymer-chain mobility leads to crystallization in the β-phase. On the contrary, upon vacuum application, a polymer is able to crystallize into another electroactive γ-phase. Furthermore, this effect is independent of the filler content, and, therefore, sample processing conditions are more determinant on phase content that the presence of a filler.

In order to quantify this effect, the crystalline phase content, the two specific bands at 766 and 833 cm^−1^ that represented the α- and γ-phases, respectively, and the method explained elsewhere were used [12]:

(5) $$F\left(\gamma \right)=\frac{X_{\gamma }}{X_{\alpha }+X_{\gamma }}=\frac{A_{\gamma }}{\left(K_{\gamma }/K_{\alpha }\right)A_{\alpha }+A_{\gamma }}$$

where *A_α_* and *A_γ_* represent the absorbencies at 766 and 833 cm^−1^ corresponding to the α- and γ-phases, *K_α_* and *K_γ_* are the absorption coefficients at the respective wavenumbers (set at 0.365 and 0.150 µm^−1^, respectively), and *X_α_* and *X_γ_* represent the degree of crystallinity of each phase. The amount of the γ-phase remained at ~90% for all the compositions, including pristine PVDF, thus confirming the previous discussion.

### 3.3. PVDF/CNC Thermal and Mechanical Properties

**Figure 4:**
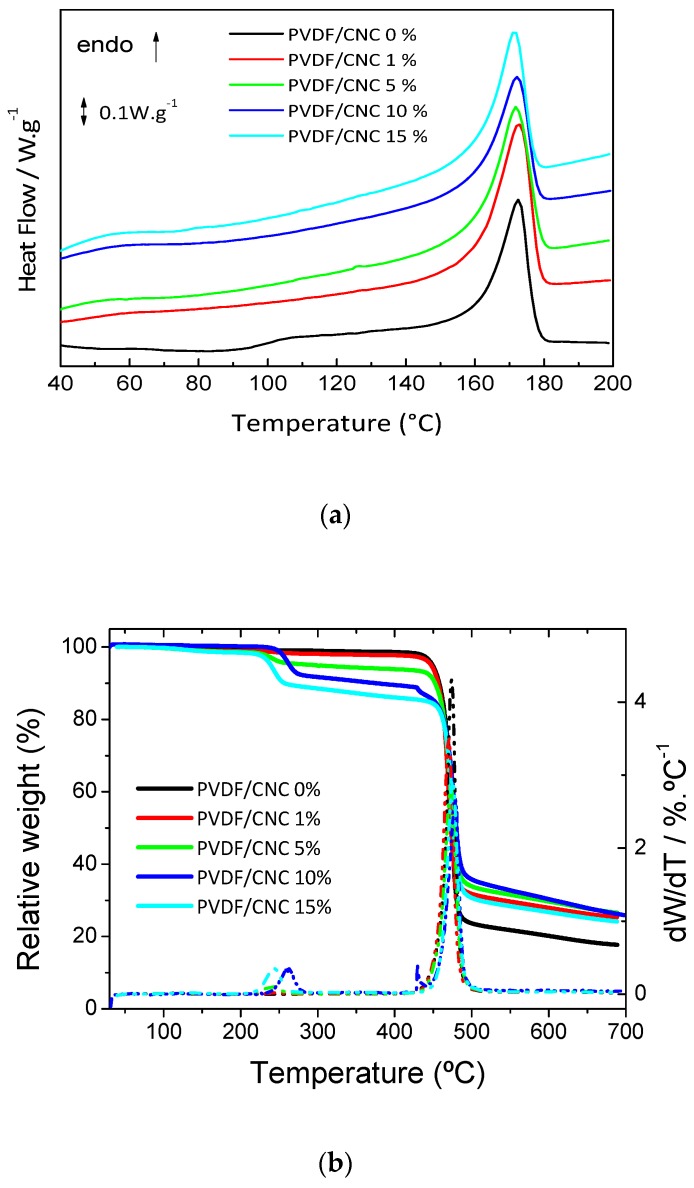
(**a**) DSC heating curves and (**b**) TGA traces of the PVDF/CNC nanocomposites.

Differential scanning calorimetry (DSC) was performed to study the occurrence of thermal transitions in the PVDF/CNC nanocomposites. Figure 4a shows the first DSC heating scans of the PVDF/CNC films that were obtained at a heating rate of 10 °C·min^−1^ (see Figure S1c for the DSC scan corresponding to the film that was dried at 60 °C under atmospheric pressure). A sharp endothermic peak was observed at a temperature of nearly 173 °C (identified as *T_m_*, melting temperature, Table 2), which was due to the melting of the crystalline domains within the material [1]. It was observed that CNC incorporation led to a slight decrease of the *T_m_* of the nanocomposites from 172.5 °C for neat PVDF up to a minimum of 171.3 °C for the 15 wt.% nanocomposite. This effect was related to the fact that CNCs act as nucleating elements, resulting in the formation of extra but smaller and slightly more imperfect crystals when compared to neat PVDF [43]. Interestingly, the heat capacity change (Δ*C_p_*) at *T_g_* was markedly reduced upon the addition of CNCs. This effect that arose from the constraining behavior of CNC surfaces on the mobility of the amorphous regions of PVDF is a typically found behavior in polymer nanocomposites and is often referred as chain confinement [44,45]. As an increase in CNC concentration is accompanied by a rise on the amount of CNC surfaces that are available to interact with PVDF chains, samples containing larger concentrations of CNCs presented a less marked Δ*C_p_*. The addition of CNCs did not substantially modify the melting temperature of the nanocomposites, at it remained at 172 ± 1 °C for all the compositions. Once the PVDF phase was determined through FTIR, it was possible to use the extent of the melting endotherm recorded during the heating DSC scan to quantitatively determine the crystalline fraction *X_c_* (%) of the prepared nanocomposites as:

(6) $$X_{c}\left(\%\right)=\frac{\Delta H_{f}}{\Delta H_{f}^{0}\times W_{m}}$$

where Δ*H_f_* is the fusion enthalpy measured during the heating scan, *W_m_* is the PVDF matrix weight fraction, and Δ*H_f_^0^* is the heat of fusion of an infinitely thick PVDF crystal (set at 104.6 J·g^−1^) [1]. The crystalline fraction present in PVDF/CNC nanocomposites is shown in Table 2. It could be observed that the crystalline fraction was affected by the amount of CNCs present in the nanocomposite. In fact, all nanocomposites showed an increasing crystalline fraction that was independent of the CNC concentrations when compared to the neat PVDF, and the highest crystalline fraction was obtained for the nanocomposite with 1 wt.%. The main reason for this was that CNCs are effective nucleating agents for PVDF crystallization [19,40]. However, at concentrations exceeding 5 wt.%, the *X_c_* increase upon CNC incorporation (in comparison with neat PVDF) was less pronounced due to the competing nucleating and constraining behavior of the CNC surfaces originated by aggregated CNC crystals. In other words, although CNC surfaces can effectively nucleate CNC crystallization, large CNC fractions do not allow polymer chains to pack into ordered large structures [19,40], thus yielding a smaller *X_c_* increase.

The study of the thermally-induced degradation in thermoplastics is of paramount relevance towards the applicability of the developed materials as the onset of the thermal degradation determines both the maximum processing temperature during conformation processes and the upper limit to service temperature. In this sense, a thermogravimetric analysis (TGA) in an N_2_ atmosphere was carried out for the PVDF/CNC films. Figure 4b shows the TGA curves (left axis, solid line) and weight loss rates or derivative thermogravimetry (DTG) curves (right axis, dotted line) of the PVDF/CNC nanocomposites. A marked effect on the thermodegradation behavior of PVDF was observed after CNC incorporation. While the thermal degradation or PVDF proceeded in a single step centered at 474 °C involving the formation of fragments arising from vinylidene monomers, dimers and oligomers [46], CNC incorporation progressively yielded a new thermodegradation process at a temperature of 243 °C. This process, which was easily observed as a step on the TGA curves and as a peak on the DTGA curves, comprised the degradation of less-thermally stable CNC fraction within the nanocomposites as a result of the depolymerization, dehydration and decomposition of cellulosic glycosyl units [47]. In any case, it should be noted that the temperature at which the maximum degradation rate was obtained, *T_peak_*, remained barely unchanged at ~474 °C, suggesting that the presence of CNCs did not catalyze thermodegradation reactions in the PVDF (Table 2). When in application, PVDF/CNC nanocomposites should be therefore limited to environments below approximately 220 °C, as further increases result in the degradation of cellulosic moieties.

**Figure 5:**
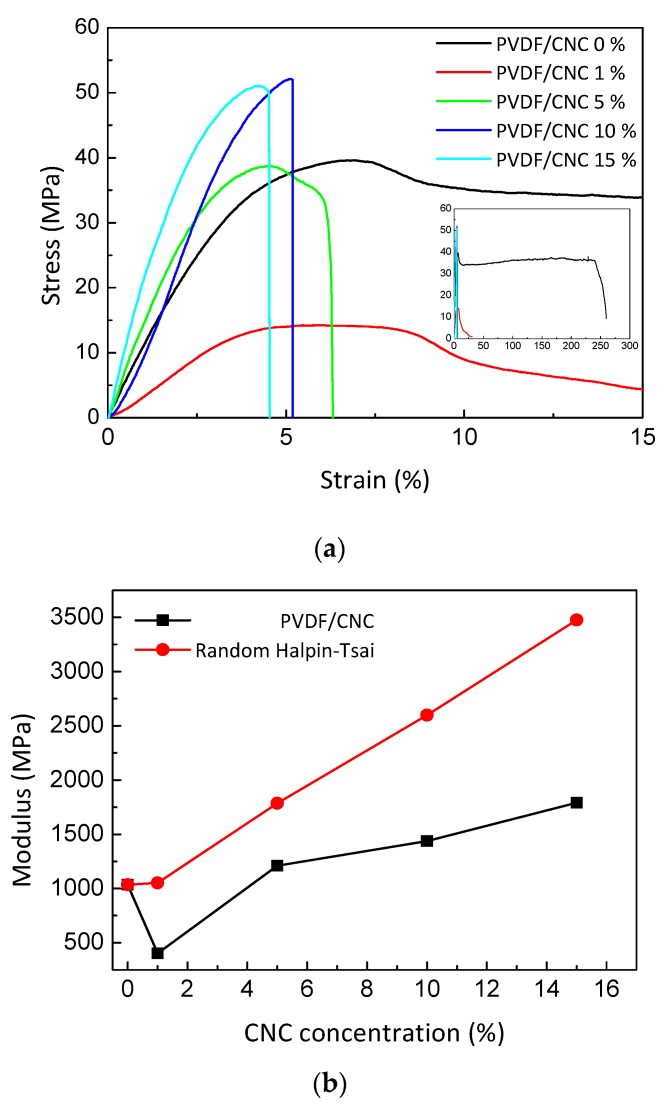
(**a**) Stress–strain tensile curves of the PVDF/CNC nanocomposites and (**b**) experimental data and fitting results according to the modified Halpin–Tsai model.

The mechanical properties of nanocomposites were evaluated by uniaxial tensile testing. Representative stress–strain curves of PVDF/CNC nanocomposites are shown in Figure 5, and the respective mechanical values (Young’s modulus (*E*), stress at yield (*σ_y_*) and strain at break (*ε_b_*)) are presented in Table 3. The neat PVDF had a ductile behavior with a stress at yield (*σ_y_*) of 39.6 MPa, a strain at yield (*σ_y_*) of 6.8%, and a strain at break (*ε_b_*) of about 250%, matching well with previously reported data and in contrast with the brittle behavior of the neat PVDF when dried at 60 °C under atmospheric pressure (see Figure S1d for the corresponding tensile stress–strain curve) [9].

The incorporation of CNCs has been shown to be accompanied by a reduction in *ε_b_* up to values as low as 4.5% for its 15 wt.% nanocomposite as similarly found in other CNC-reinforced polymeric systems [40,48]. This is a commonly found effect in polymer nanocomposites and is ascribed to a combination of a poor interfacial adhesion between the filler and the matrix and CNC aggregation, which yields inefficient local stress transference [49,50]. Here, this continuous embrittlement upon CNC incorporation was accompanied by a marked increase in both Young´s modulus (*E*) from 1035 ± 170 MPa for neat PVDF to 1662 ± 47 MPa for its 15% nanocomposite counterpart. In any case, it is worth noting that at low concentrations, e.g., 1 and 5 wt.%, CNC incorporation notably decreased both *E* and *σ_y_* up to a minimum of 427 ± 63 and 14.3 MPa, respectively, indicating a poor interfacial compatibility between the hydrophobic character of PVDF and the highly hydrophilic CNCs [51,52]. The fact that the Young´s modulus increased by only 60% with the presence of 15 wt.% CNC also suggested a poor interfacial compatibility with a possible CNC aggregation. It was also noticed that although the *E* modulus increased upon CNC addition due to the presence of stiff CNCs, tensile strength slightly decreased at a concentration of 15 wt.% CNC. This effect arose from the weak interfacial compatibility between CNC and PVDF, which effectively transferred stresses at low strains (therefore, *E* increased) but failed to effectively reinforce the whole system at high strains. Overall, the interfacial compatibility could be improved by surface grafting of CNCs before their introduction into the PVDF, as the grafted chains could create a co-continuous phase for the efficient transfer of local stresses within the bulk material.

Theoretical models are gaining importance in polymer science as reliable tools to save time and help towards successful approaches to customize final composite properties. Among all the available possibilities, the Halpin–Tsai model was used here as opposed to other micromechanical models because, in spite of its limitations, its semi-empirical friendly character has served to date to successfully predict the elastic modulus of CNC-based nanocomposites [53,54,55]. Therefore, experimental results were compared with theoretical predictions according to the modified Halpin–Tsai model [56]:

(7) $$\frac{E_{c}}{E_{m}}=\left(\frac{3}{8}\right)\left(\frac{1+2\rho \eta_{L}V_{CNC}}{1-\eta_{L}V_{CNC}}\right)+\left(\frac{5}{8}\right)\left(\frac{1+2\eta_{T}V_{CNC}}{1-\eta_{T}V_{CNC}}\right),$$

(8) $$\eta_{L}=\frac{E_{r}-1}{E_{r}+2\rho },\;\eta_{T}=\frac{E_{r}-1}{E_{r}+2},$$

where *E_c_* and *E_m_* is the Young´s modulus of the nanocomposite and PVDF matrix, respectively, *ρ* is the CNC aspect ratio, *V_CNC_* is its volume fraction, and *E_R_* is the ratio between the Young´s modulus of CNCs and the PVDF matrix. The CNC volume fractions were calculated from the weight fraction and densities of PVDF and CNC (1.78 g·cm^−3^ for PVDF and 1.6 g·cm^−3^ for CNC [57]), while the Young’s modulus of the CNCs was computed to be 105 GPa [58]. The comparison between the predicted and experimentally obtained *E* values according to Equation (7) is shown in Figure 5b. It was observed that the experimental values remained below the predicted *E* for all the studied compositions. This effective modulus of elasticity over-prediction, especially for the 1 wt.% concentration, indicates an ineffective stress transfer across the PVDF–CNC interfaces [40,45]. It was seen that at very low concentrations, the PVDF–CNC interfaces were weak points where concentration stresses were formed upon uniaxial tensile stretching, thus yielding a marked decrease of *E*.

### 3.4. PVDF/CNC Dielectric Properties

**Figure 6:**
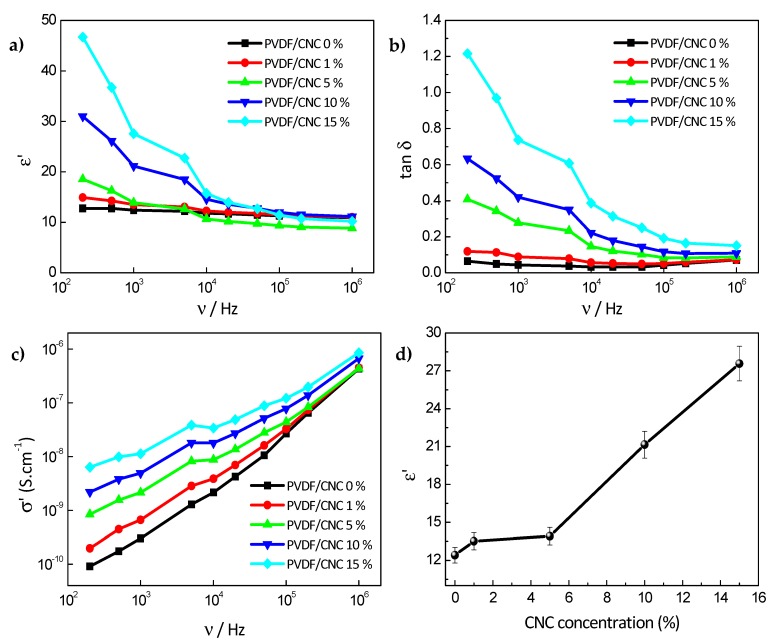
Dielectric spectroscopy results. (**a**) Frequency dependence of the real part of the permittivity, (**b**) tangent loss, (**c**) real part of the dielectric constant and dielectric loss tangent at 1 KHz as a function of CNC concentration, and (**d**) concentration dependence of the real part of the permittivity for PVDF/CNC nanocomposites.

Dielectric spectroscopy was conducted to understand the effect of CNC incorporation on the dielectric response of the PVDF/CNC nanocomposites. Figure 6a reports the influence of the CNC concentration on the real part of the permittivity in the range of 10^2^–10^6^ Hz, while the dielectric loss tangent (*tan δ = ε′′/ε′*) for the PVDF/CNC nanocomposites is displayed in Figure 6b. For the sake of comparison, Figure 6c shows the real dielectric constant and the dielectric loss tangent at room temperature of the PVDF/CNC nanocomposites at 200 Hz as a function of CNC concentration. It was observed that the dielectric constant (*ε′*) decreased with increasing frequency due to the slower dynamic of the dipoles with respect to the applied electric field [59]. The neat PVDF film presented a dielectric constant of 13 at 200 Hz, while the CNC incorporation markedly increased the *ε′* of nanocomposites, in particular at low frequencies. For instance, at 200 Hz, *ε′* increased by 3.6 times up to 47 for the 15 wt.% CNC nanocomposite. These results suggest that CNCs are able to increase dielectric response through Maxwell–Wagner–Sillars interfacial polarization contributions and local ionic conductivity in PVDF nanocomposites [60]. This increase was notably larger than that reported for PVDF/carbon nanofibers [9], highlighting the efficiency of CNCs as additives to tune the functional properties of PVDF. For instance, high-dielectric constant polymeric membranes with high ionic conductivity may be used as lithium ion battery (LIB) separators due to both their enhanced ionic conductivity and increased charge carrier concentration [61]. For a given frequency, the dielectric loss tangent increases with CNC concentration, indicating the greater presence of interfacial polarization and electronic dipole polarization, leading to significant energy dissipation [62,63]. Similarly, the fact that the AC conductivity (*σ*) shown in Figure 6d increased with both CNC loading and frequency may be explained in terms of the shorter distance between the carriers upon CNC incorporation, which enabled charge flow via a hopping mechanism [64]. Similar results have also been reported after the incorporation of cellulose nanofibers and cyanoethylcellulose into PVDF [61].

The AC conductivity behavior shown in Figure 6d was related to the hopping transport of the localized charge carriers, which can increase with CNC presence, as described by [65]:

(9) $$\sigma^{\prime }\left(\omega \right)\propto \omega^{n},n\leq 1,$$

where ω is the angular frequency and n ranges between 0 < *n* < 1, thus characterizing the hopping conduction and representing the degree of the interaction between the mobile ions and the surrounding environment [66]. The n exponent extracted from the slope through Equation (8) and Figure 6c for all PVDF/CNC nanocomposites is shown in Table 4.

It was observed that n values ranged between 0.5 to 1 and increased with CNC concentration due to the presence of negatively charged CNCs, which are able to increase charge carriers in localized states and the excitation of charge carriers to upper states in the conduction band [67].

## 4. Conclusions

This work reports on the fabrication of nanocomposite materials based on PVDF and CNCs. The XRD and FTIR results revealed the formation of the polar and electrically active γ-phase of PVDF for all the compositions, while high-magnification SEM observations showed no traces of CNC bundles. It was found that the *X_c_* crystalline fraction in PVDF increased at low CNC concentrations, yielding materials with a larger electroactive γ-phase. This effect was attributed to the preparation conditions, mainly vacuum drying. The Young´s modulus of nanocomposites first decayed at low concentrations to further increase from 1035 MPa for neat PVDF to 1790 MPa for its 15% nanocomposite. This behavior was ascribed to a generally poor interfacial compatibility between the hydrophobic character of PVDF and the highly hydrophilic CNCs.

Dielectric spectroscopy showed a marked increase on the real dielectric constant of nanocomposites due to an enhanced ionic conductivity and charge carrier concentration provided by CNCs. Moreover, AC conductivity increased with CNC loading due to the shorter distance between the carriers upon CNC incorporation, which enabled charge flow via a hopping mechanism. Overall, this work provides novel pathways for the development of γ-phase PVDF with a high dielectric constant and improved ionic conductivity, thus showing a large potential to develop cellulose-based electroactive materials for multifunctional applications.

## Figures and Tables

**Table 1:** Specific vibration modes characteristics of PVDF and CNC.

Wavenumber (cm^−1^)	Material	Vibrational Mode
897	CNC	C–O–C asymmetric stretching
1160	CNC	C–O–C bending
1337	CNC	C–O–H bending
3650–3200	CNC	O–H stretching
766	PVDF	α-phase
796	PVDF	α-phase
812	PVDF	γ-phase
833	PVDF	γ-phase
838	PVDF	γ-phase
840	PVDF	β-phase
855	PVDF	α-phase
976	PVDF	α-phase
1234	PVDF	γ-phase
1275	PVDF	β-phase

**Table 2:** Crystalline fraction (*X_c_*), melting temperature (*T_m_*), and maximum degradation temperature (*T_dpeak_*) for the PVDF/CNC nanocomposites.

Sample Name	*X_c_* (%)	*T_m_* (°C)	*T_dpeak_* (°C)
PVDF/CNC 0 wt.%	38 ± 2	173	473
PVDF/CNC 1 wt.%	53 ± 4	173	470
PVDF/CNC 5 wt.%	48 ± 2	172	471
PVDF/CNC 10 wt.%	43 ± 2	172	478
PVDF/CNC 15 wt.%	48 ± 3	171	470

**Table 3:** Mechanical parameters for the prepared PVDF/CNC nanocomposites.

SampleN	*E* (MPa)	*σ_y_* (MPa)	*ε_b_* (%)
PVDF/CNC 0 wt.%	1035 ± 170	39.6	250
PVDF/CNC 1 wt.%	427 ± 63	14.3	9.5
PVDF/CNC 5 wt.%	1249± 166	39.2	6.3
PVDF/CNC 10 wt.%	1410± 123	52.7	5.2
PVDF/CNC 15 wt.%	1662 ± 47	51.1	4.5

**Table 4:** *n* exponent for all PVDF/CNC nanocomposites.

Sample Name	*n*
PVDF/CNC 0 wt.%	0.5
PVDF/CNC 1 wt.%	0.6
PVDF/CNC 5 wt.%	0.7
PVDF/CNC 10 wt.%	0.9
PVDF/CNC 15 wt.%	1

## Supplementary Materials

The following are available online at https://www.mdpi.com/1996-1944/13/3/743/s1, Figure S1: (a) Representative SEM image, (b) FTIR spectra, (c) DSC heating scan, and (d) stress–strain tensile curve for PVDF obtained upon drying at 60 °C at atmospheric pressure. Figure S2: Enlarged FTIR spectrum highlighting the band located at 1033 cm^−1^ corresponding to sulphate half-ester groups in CNCs.
