# Supplementary material for: Electroactive γ-Phase, Enhanced Thermal and Mechanical Properties and High Ionic Conductivity Response of Poly (Vinylidene Fluoride)/Cellulose Nanocrystal Hybrid Nanocomposites
Source: Materials (Basel). 2020 Feb 6;13(3):743. doi: 10.3390/ma13030743 (PMC7040804; doi:10.3390/ma13030743)
Supplement: Supplementary file 1 [file materials-13-00743-s001.pdf]

materials

Supplementary Materials

Electroactive γ-Phase, Enhanced Thermal and Mechanical Properties and High Ionic Conductivity Response of Poly (Vinylidene Fluoride)/Cellulose Nanocrystal Hybrid Nanocomposites

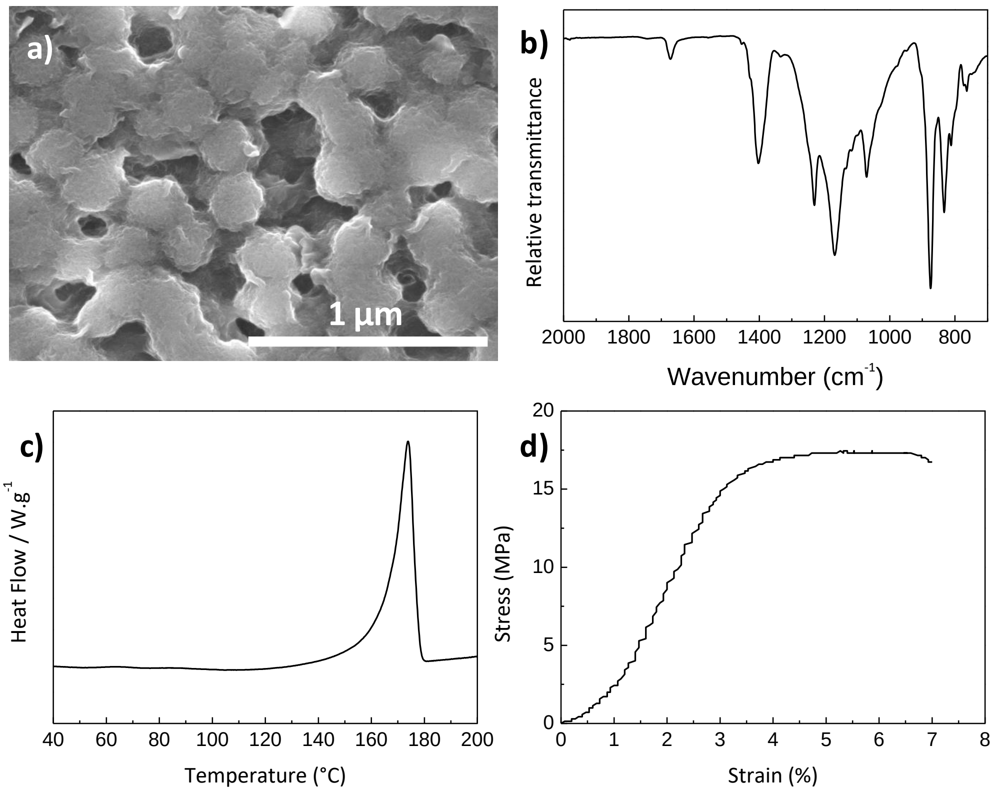

Figure S1. (**a**) Representative SEM image; (**b**) FTIR spectra; (**c**) DSC heating scan and (**d**) stress-strain tensile curve for PVDF obtained upon drying at 60 °C at atmospheric pressure.

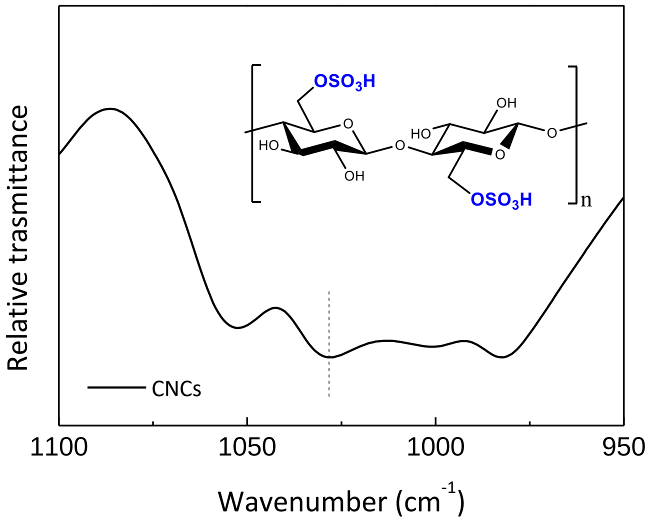

Figure S2. Enlarged FTIR spectrum highlighting the band located at 1033 cm^{-1} corresponding to sulphate half-ester groups in CNCs.
